# Alterations of brain activity in multiple system atrophy patients with freezing of gait: A resting-state fMRI study

**DOI:** 10.3389/fnins.2022.954332

**Published:** 2022-08-16

**Authors:** Yilin Cheng, Huaguang Yang, Weiyin Vivian Liu, Zhi Wen, Jun Chen

**Affiliations:** ^1^Department of Radiology, Renmin Hospital of Wuhan University, Wuhan, China; ^2^MR Research, GE Healthcare, Beijing, China

**Keywords:** multiple system atrophy, freezing of gait, degree centrality, functional connectivity, fMRI

## Abstract

**Background:**

Freezing of gait (FOG) in multiple system atrophy (MSA) is characterized by a higher risk of falls and a reduced quality of life; however, the mechanisms underlying these effects have yet to be identified by neuroimaging. The aim of this study was to investigate the differences in functional network when compared between MSA patients with and without freezing.

**Methods:**

Degree centrality (DC) based on the resting-state functional magnetic resonance imaging was computed in 65 patients with MSA and 36 healthy controls. Brain regions with statistically different DC values between groups were selected as seed points for a second seed-based functional connectivity (FC) analysis. The relationships between brain activity (DC and FC alterations) and the severity of freezing symptoms were then investigated in the two groups of patients with MSA.

**Results:**

Compared to MSA patients without FOG symptoms (MSA-nFOG), patients with MSA-FOG showed an increased DC in the left middle temporal gyrus but a reduced DC in the right superior pole temporal gyrus, left anterior cingulum cortex, left thalamus, and right middle frontal gyrus. Furthermore, in patients with MSA-FOG, the DC in the left thalamus was negatively correlated with FOG scores. Using the left thalamus as a seed, secondary seed-based functional connectivity analysis revealed that patients with MSA-FOG commonly showed the left thalamus-based FC abnormalities in regions related to cognition and emotion. In contrast to the patients with MSA-nFOG, patients with MSA-FOG showed an increased FC between the left thalamus and the left middle temporal gyrus (MTG), right inferior parietal lobule (IPL), bilateral cerebellum_8, and left precuneus.

**Conclusion:**

Freezing of gait is associated with centrality of the impaired thalamus network. Abnormal FC between the thalamus and left MTG, right IPL, bilateral cerebellum_8, and left precuneus was involved in FOG. These results provide new insight into the pathophysiological mechanism of FOG in MSA.

## Introduction

Multiple system atrophy (MSA) is a sporadic and progressive neurodegenerative disease that is manifested by prominent autonomic nerve function symptoms such as urinary retention and orthostatic hypotension (Fanciulli et al., 2019). Freezing of gait (FOG) is a debilitating symptom defined as a brief, episodic absence or marked reduction in forward progression of the feet despite the intention to walk (Nutt et al., 2011). Epidemiological studies have shown that approximately 76% of patients with MSA experience a frozen gait, thus leading to an increased risk of fall and a reduced quality of life (Gurevich and Giladi, 2003). Although the incidence of MSA with frozen symptoms gradually increases with disease progression, not all patients with MSA develop FOG (Factor, 2008). Therefore, identifying FOG-driven alterations in the associated pathophysiological mechanism may help to provide a better understanding of this disabling symptom and help clinicians to provide appropriate interventions.

Functional magnetic resonance imaging (fMRI) techniques appear to be ideally suited to investigate the neuropathology of FOG. It was previously found that regional homogeneity values in the left supplementary motor area and the left superior frontal region were significantly reduced in patients with Parkinson’s disease (PD) and FOG when compared to PD patients without FOG (Zhou et al., 2018). Patients with PD-FOG showed wider alterations in the resting-state network than patients with non-FOG; furthermore, freezing severity was modulated by the left superior temporal gyrus in patients with PD (Liu et al., 2019). Patients with PD-FOG also showed more significant reductions in the volume of the cortical gray matter in the parietal lobe and subcortical caudate areas (Kostic et al., 2012; Herman et al., 2014) and increased damage in the white matter of the corticopontine-cerebellar pathways (Wang et al., 2016) than PD patients without FOG. Taken together, these data indicate that structural integrity and the potential functional disconnection of cortical regions and the subcortical nucleus are related to FOG in PD. However, FOG symptoms are more common in patients with MSA than in patients with PD (Factor, 2008). To date, such investigations have not been conducted in patients with MSA.

To explore the impairments in the functional network of MSA patients with FOG symptoms, we first used data-driven research methods to analyze DC to detect the hub alteration in the FOG-associated network in patients with MSA. The advantage of this strategy is to avoid bias caused by seed point selection. In the second step, we used a secondary seed-based functional connection method to select altered DC brain areas as seeds to investigate the cortical hub-based circuit in the regulation of FOG in patients with MSA from point to surface. We hypothesized that patients with MSA-FOG would have impairments in cortical and subcortical structure and function and that these impairments would reflect the severity of FOG in patients with MSA. By selecting these brain regions as seed points, we further hypothesized that these specific brain regions would be used mainly to cooperate or antagonize the abnormalities of other brain networks participating in the regulation of FOG symptoms in patients with MSA.

## Materials and methods

### Subjects

In total, 65 patients with MSA and 36 healthy controls (HCs) were recruited from the Department of Neurology at Renmin Hospital of Wuhan University. All participants were Han Chinese and right-handed. The inclusion criteria were patients who were diagnosed with probable or possible MSA (H-Y stage score ≤ 4, Mini-Mental State Examination, MMSE score ≥ 25) by a movement disorder specialist according to the 2008 Second Edition MSA Diagnostic Criteria (Gilman et al., 2008). Patients were excluded if they had a history of other neurological diseases or any predominant physical diseases. This study, involving human participants, was reviewed and approved by the Renmin Hospital of Wuhan University Ethics Committee. The patients provided their written informed consent to participate in this study.

All patients experienced a 12-h drug washout before examination (clinical motor and non-motor scale evaluation and MRI scans). MSA disease severity and stage was scored using the Unified Multiple System Atrophy Rating Scale III (UMSARS III) and the Hoehn and Yahr (H-Y) stage, respectively. Global cognitive function and mood of the patients with MSA were assessed by the Mini-Mental State Examination (MMSE), and the 24-item Hamilton Depression Rating Scale (HAMD-24), respectively.

Freezing episodes were observed by experienced neurologists during hospital visits, self-reported by patients, or described by their caregivers. Patients were diagnosed as the so-called freezers who had a positive FOG according to items 1 and 3 on the FOG questionnaire (FOG-Q) (Giladi et al., 2009). If the patients, or their family members, could not understand the definition of FOG, a description or a possible FOG subtype was determined by neurologists during hospital visits. According to the FOG-Q scale, there were 36 patients with MSA-FOG and 29 MSA patients without FOG (MSA-nFOG). Patients with MSA were follow-up patients who were taking levodopa drugs and were classified as the “OFF-FOG” group.

### MRI acquisition

All subjects underwent an MRI examination with a 3T MRI scanner (GE Discovery MR750W; GE Healthcare, United States) using a 16-channel head coil. Participants completed a high-resolution, three-dimensional, sagittal magnetization gradient echo imaging sequence (3D-T1) with the following acquisition parameters: repetition time/echo time = 8.5/3.3, matrix = 256 × 256, flip angle = 12°, voxel size = 1.0 mm × 1.0 mm × 1.0 mm, slice thickness = 1 mm without slice gap, FOV = 256 mm^2^× 256 mm^2^, and slice number = 180. The participants also received a blood oxygen level-dependent (BOLD) sequence scan using the following parameters: repetition time = 2,000 ms, echo time = 25 ms, flip angle = 90°, slice number = 40, slice thickness = 3 mm without slice gap, FOV = 240 mm × 240 mm, matrix size = 64 × 64, and voxel size = 3 mm × 3 mm × 3 mm.

### Degree centrality and functional connectivity analysis

#### Degree centrality analysis

Resting-state functional MRI data processing and analysis were performed using DPABI software (Data Processing and Analysis for Brain Imaging, version 6.0^1^). The first 10 time points were discarded due to non-homogeneity of magnetic resonance field strength and a noise adaptation period for subjects. The remaining images were slice timed and realigned; subjects with head movement greater than 0 mm or 2.5° in any direction were excluded from subsequent analysis. The remaining data were then normalized into a Montreal Neurological Institute template standard space of 3 mm^3^× 3 mm^3^ × 3 mm^3^ (DARTEL technique) (Ashburner, 2007). For the regression of nuisance covariates, we applied Friston-24 parameters and removed signals from the white matter and cerebrospinal fluid. Subsequently, linear trends were removed and band-pass filtered (0.01–0.08 Hz). Any volume with a frame-wise displacement value exceeding 0.5 images was excluded to remove nuisance covariate effects.

DPABI software was used to analyze the non-smoothened and preprocessed fMRI data, so that we could calculate density correlations. Pearson’s correlation method was utilized to correlate the time series of each voxel and those of all other voxels to create a whole brain DC map and obtain the connection matrix of correlation coefficients for the whole brain. *R* > 0.25 was selected as the threshold value to eliminate low time correlations caused by signal noise (Wang et al., 2018; Yang et al., 2020). Then, 6 mm × 6 mm × 6 mm full width at half maximum Gaussian kernels were used to spatially smoothen all individual zDC maps. Only positively weighted Pearson’s correlation coefficients were considered as the number of functional connections at the individual level because of the uncertainty arising when interpreting negative values.

#### Secondary seed-based functional connectivity analysis

For seed-based FC calculation, the preprocessed image was further smoothened with a 6 mm^3^ Gaussian kernel. We selected significant DC alterations associated with the FOG-Q scale as hubs between the MSA-FOG and patients with MSA-nFOG. Specifically, we extracted the reference time series from the DC results by averaging the time series of every voxel in seed regions and conducted further correlation analyses between the time courses and time series of voxels inside and outside of the seed regions in the entire brain. The correlation coefficients were then converted into *Z*-values using Fisher’s r-to-z transformation. In addition, we further analyzed the FC map of the spherical region within a 3-mm radius that covered the peak group difference between DC values to eliminate seed selection-related influences (refer to Supplementary material 1).

### Statistical analysis

Statistical Package for the Social Sciences (SPSS) version 22.0 software (SPSS Inc., Chicago, IL, United States) was used to compare demographic and clinical variables between groups. Demographic data were presented as mean ± standard deviation (SD) for continuous variables. The independent sample *t*-test and Kruskal–Wallis test, or analysis of variance (ANOVA) followed by Tukey’s test for normally distributed data or the Bonferroni test for non-normally distributed data, were used for cross-group comparisons of quantitative variables. The chi-squared test was used to compare categorical variables. We set the threshold at *p* < 0.05 for the level of significance.

The SPM statistical analysis module was used for neuroimaging data. One-way ANOVA was used to identify DC differences among the MSA-FOG, MSA-nFOG, and HC groups by controlling confounding factors including age, gender, MMSE, Unified Multiple System Atrophy Rating Scale (UMSARS), and HAMD score covariates; then, significant different brain areas were extracted as a mask. Next, *post hoc* and multiple comparison corrections (FDR correction, *p* < 0.05) were performed to identify the differences between groups within the masks and in the whole brain.

To identify the relationship between brain activity and the severity of FOG, Pearson’s correlation was computed between the DC values and FOGQ scores. Brain regions with significantly different FOG-related DC values were selected as seeds for a secondary seed-based FC analysis.

## Results

### Clinical and neuropsychological characteristics

The demographic and clinical characteristics of the MSA-FOG, MSA-nFOG, and HC groups are shown in Table 1. There were no significant differences between the three groups in terms of age, gender, education, and MMSE scores. Furthermore, there were no significant differences between the MSA-FOG and MSA-nFOG groups with regard to clinical subtypes, H-Y grade, and the UMSARS score.

**TABLE 1:** Demographic and clinical characteristics.

Characteristics (mean ± SD)	Control (*n* = 36)	MSA-FOG (*n* = 36)	MSA-nFOG (*n* = 29)	F/χ^2^	*P*-value
Age (years)	63.25 ± 3.34	64.50 ± 7.31	62.83 ± 8.32	0.59	0.56
Gender (male, female)	22:14	16:20	15:14	1.20	0.28
Education	11.72 ± 2.74	10.14 ± 3.08	10.48 ± 3.78	2.43	0.09
Disease duration	N	4.17 ± 1.96	2.67 ± 1.32	2.32	0.13
UMSARS score	N	37.51 ± 14.10	29.67 ± 15.91	0.67	0.42
Hoehn and Yahr	N	2.94 ± 0.98	2.40 ± 0.76	0.80	0.38
LEED (mg/day)	N	548.60 ± 308.14	403.02 ± 230.45	2.43	0.12
Clinical phenotype (P/C)	N	16:20	16:13	2.04	0.36
MMSE score	28.67 ± 1.07	28.31 ± 0.92	28.52 ± 1.09	1.13	0.328
HAMD-24 score	1.69 ± 2.35	2.50 ± 1.82	2.21 ± 1.50	1.57	0.214

SD, standard deviation; MSA, multiple system atrophy; MSA-FOG, multiple system atrophy with freezing of gait symptoms; MSA-nFOG, multiple system atrophy without freezing of gait symptoms; HCs, healthy controls; UMSARS, Unified Multiple System Atrophy Rating Scale; LEED, levodopa equivalent dose; P/C, Parkinson’s type/cerebellar type; MMSE, Mini-Mental State Examination; HAMD-24, 24 items Hamilton Depression Scale; p < 0.05 was considered statistically significant.

### Degree centrality analysis

**FIGURE 1:**
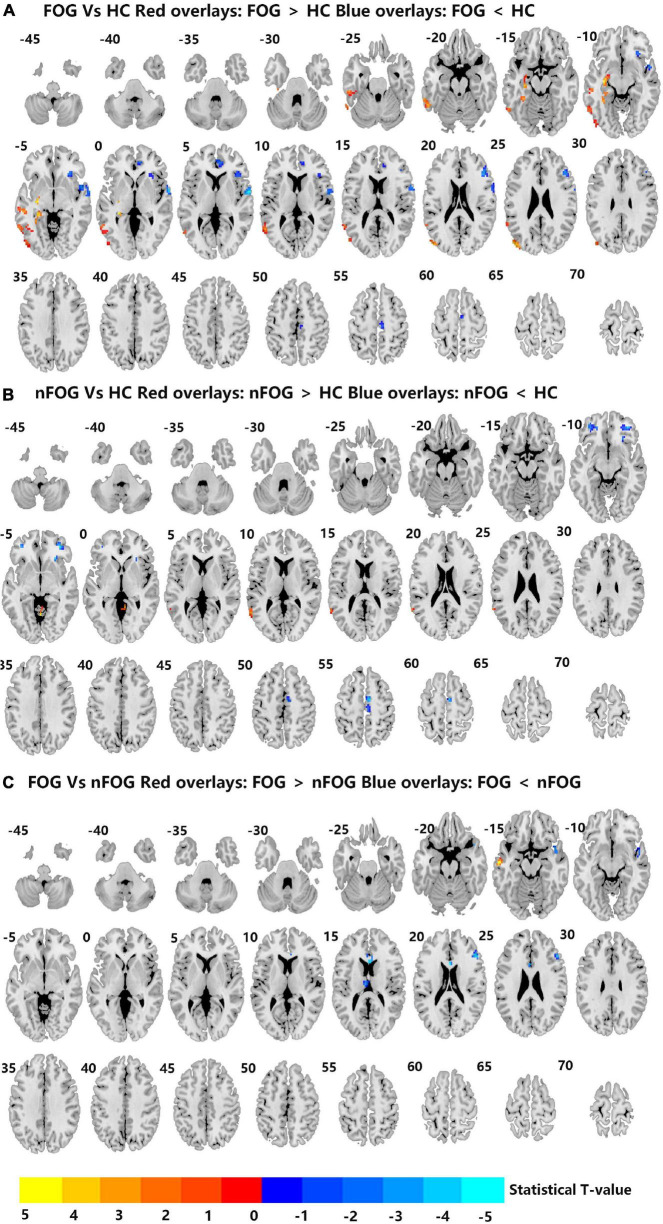
**(A–C)** Differences in degree centrality among the MSA-FOG, MSA-nFOG, and HC groups. The threshold value was set as an FDR-corrected *p* < 0.05. MSA-FOG, multiple system atrophy with freezing of gait; MSA-nFOG, multiple system atrophy without freezing of gait symptoms; HCs, healthy controls.

Compared to the HC group, the MSA-FOG group showed an increased DC in the left inferior, middle and superior temporal gyrus, the left middle occipital gyrus, and the left hippocampus but a reduced DC in the right inferior orbit frontal gyrus, right superior temporal gyrus, right anterior cingulum cortex, and right medial frontal gyrus. The patients with MSA-nFOG had an increased DC in the cerebellum vermis IV/V and left middle temporal gyrus but a reduced DC in the right inferior orbit frontal gyrus and bilateral middle orbit frontal gyrus. Compared to the MSA-nFOG group, the MSA-FOG group showed an increased DC in the left middle temporal gyrus but a decreased DC in the right superior pole temporal gyrus, left anterior cingulum cortex, left thalamus, and right middle frontal gyrus (Figure 1 and Table 2).

**TABLE 2:** Brain area differences in degree centrality between patients with MSA and HC.

				Peak MNI co-ordinate			
Brain regions	Hem	Cluster	BA	*X*	*y*	*Z*	*T*-value
**MSA-FOG vs. HC**							
Inferior temporal gyrus	L	39	20	−54	−27	−30	4.10
Middle temporal gyrus	L	179	21	−63	−54	12	3.89
Hippocampus	L	47	37	−30	−33	−3	4.55
Superior temporal gyrus	L	20	NA	51	9	−6	−4.27
Inferior orbit frontal gyrus	R	37	47	27	27	−6	−4.14
Superior temporal gyrus	R	93	48	63	0	3	−5.74
Anterior cingulum cortex	R	36	10	9	45	3	−4.10
Middle occipital gyrus	L	46	NA	−48	−81	24	4.21
Medial frontal gyrus	R	23	6	6	−9	60	−3.45
**MSA-nFOG vs. HC**							
Inferior orbit frontal gyrus	R	22	47	27	27	−6	−4.04
Middle orbit frontal gyrus	L	25	47	−36	45	−6	−3.74
Middle orbit frontal gyrus	R	28	47	36	45	−6	−4.47
Cerebellum Vermis IV and V	NA	21	NA	0	−60	6	3.62
Middle temporal gyrus	L	28	37	−60	−57	9	3.19
**MSA-FOG vs. MSA-n FOG**							
Superior pole temporal gyrus	R	25	48	48	6	−12	−3.59
Middle temporal gyrus	L	19	21	−60	−12	−15	3.87
Anterior cingulum cortex	L	35	NA	0	18	18	−3.87
Thalamus	L	20	NA	−6	−15	15	−3.31
Middle frontal gyrus	R	25	45	48	27	21	−3.66

A negative T-value represents decreased degree in MSA group. MSA-FOG, MSA-nFOG, multiple system atrophy patients with and without freezing of gait symptoms. BA, Brodmann area. L, R, left and right.

### Seed-based functional connectivity analysis

**FIGURE 2:**
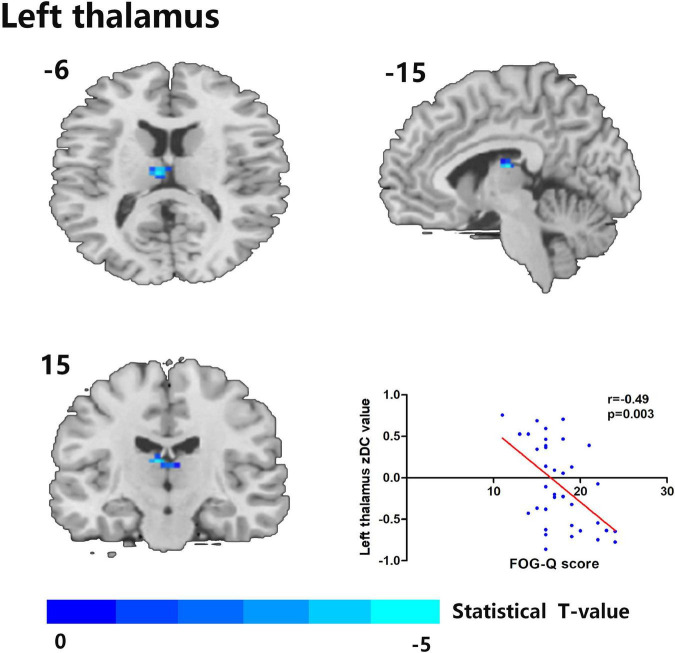
The left thalamus was the only degree centrality (DC)-altered brain area in correlation with FOG-Q scores between MSA-FOG and MSA-nFOG groups. Scatter plot showed a negative correlation between FOG-Q scores and the left thalamus zDC values in patients with MSA-FOG.

**FIGURE 3:**
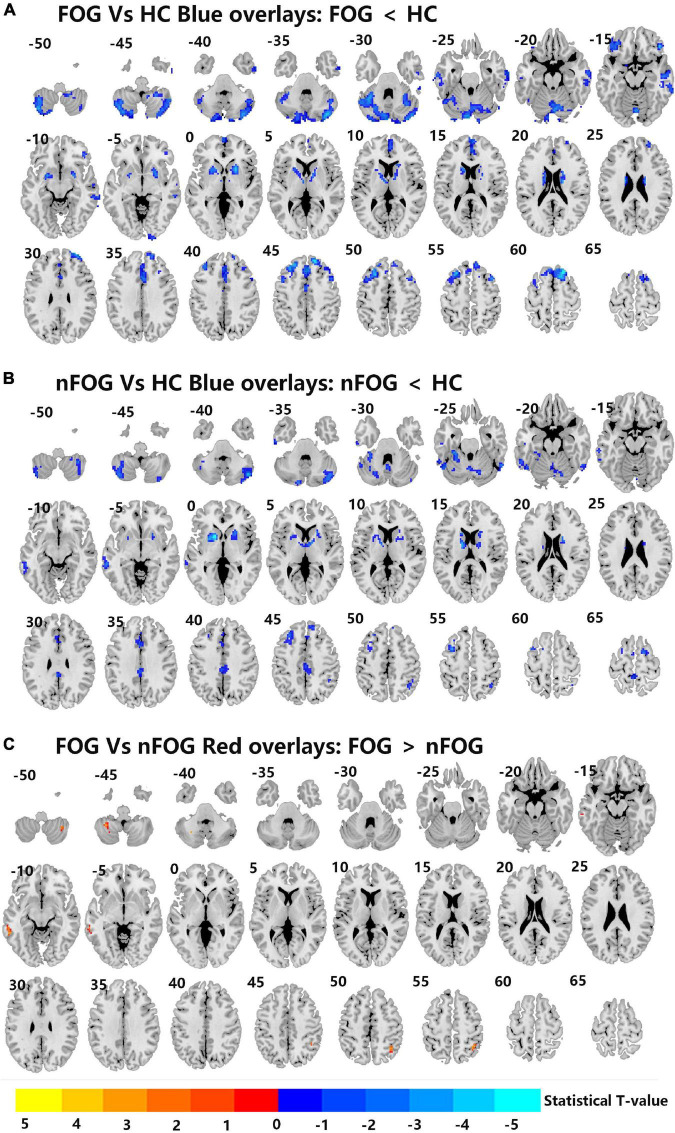
**(A–C)** Differences in left thalamus dominated FC among MSA-FOG, MSA-nFOG, and HCs using *post hoc* correction for two-sample *t*-test (FDR-corrected *p* = 0.05).

Compared to the HC group, the MSA-FOG group showed a decreased thalamus-based FC in the bilateral MTG, right hippocampus, right insula, right inferior frontal cortex, and right calcarine. In contrast, the MSA-nFOG group showed a decreased thalamus-based FC in the inferior and middle temporal gyrus. Direct comparison of the MSA-FOG and MSA-nFOG groups revealed thalamus-based FC in the left middle temporal gyrus, right inferior parietal lobule, bilateral cerebellum_8, and the left precuneus (Figures 2, 3 and Table 3).

**TABLE 3:** Brain area differences in right thalamus dominated FC network between patients with MSA and HC.

				Peak MNI co-ordinate			
Brain regions	Hem	Cluster	BA	*x*	*y*	*Z*	*T*-value
**MSA-FOG vs. HC**							
Cerebellum crus2	L	1536	NA	42	−78	−33	−5.56
Cerebellum_8	R	60	NA	9	−48	−48	−4.66
Inferior temporal gyrus	R	39	NA	57	−3	−42	−4.13
Inferior temporal gyrus	L	33	20	−60	−12	−24	−4.03
Middle temporal gyrus	R	189	20	63	−33	−12	−4.67
Inferior frontal gyrus	L	63	11	−30	42	−15	−4.65
Putamen	L	152	48	−24	3	−9	−5.01
Medial frontal gyrus	R	116	NA	0	63	18	−4.85
Putamen	R	159	11	21	12	−3	−5.41
Superior frontal gyrus	R	643	8	18	27	60	−5.94
**MSA-nFOG vs. HC**							
Cerebellum_Crus2	L	121	NA	−39	−72	−48	−4.92
Cerebellum_8	R	69	NA	33	−48	−54	−5.93
Cerebellum_Crus2	R	126	NA	45	−69	−39	−5.83
Inferior temporal gyrus	L	23	20	−57	−15	−33	−4.58
Cerebellum_6	L	95	NA	−30	−33	−24	−5.29
Cerebellum_6	R	65	NA	15	−66	−24	−4.80
Inferior temporal gyrus	R	30	37	57	−54	−24	−4.89
Middle temporal gyrus	L	72	21	−63	−36	−6	−4.80
Putamen	L	291	NA	−15	9	0	−6.75
Middle cingulum cortex	L	110	23	−3	−30	36	−4.57
Middle frontal gyrus	L	61	9	−30	30	45	−5.06
Inferior parietal lobule	R	30	40	39	−54	51	−4.63
Precuneus	L	28	5	−3	−39	66	−4.69
Superior frontal gyrus	R	30	6	18	−3	69	−4.80
**MSA-FOG vs. MSA-nFOG**							
Middle temporal gyrus	L	40	20	−57	−45	−9	3.50
Inferior parietal lobule	R	29	40	39	−54	51	3.54
Cerebellum_8	R	19	NA	39	−48	−57	4.00
Cerebellum_8	L	21	NA	−33	−48	−45	3.48
Precuneus	L	19	5	−6	−42	60	3.28

A negative T-value represents decreased right thalamus dominated FC in MSA group. MSA-FOG, MSA-nFOG, multiple system atrophy patients with and without freezing of gait symptoms; BA, Brodmann area; L, R, left and right.

### Correlation between degree centrality and seed-based functional connectivity changes with depression scores in the multiple system atrophy group

Cerebral areas of the zDC results between the MSA-FOG group and MSA-nFOG group were used to conduct correlation analysis; only the left thalamus zDC was shown to be related to the clinical FOGQ score (Figure 2). Then, using the left thalamus as a seed, we identified brain regions showing FC alterations between the MSA-FOG group and the MSA-nFOG group; none of the resulting areas (left middle temporary gyrus, right inferior parietal lobe, bilateral cerebellum_8, and left precuneus) were significantly correlated with FOGQ scores (Figure 2).

## Discussion

Freezing of gait is a common and disabling symptom in patients with MSA (Gurevich and Giladi, 2003). As the first step in our study, DC values were used to identify the differences of hubs in resting-state fMRI among patients with MSA-FOG, patients with MSA-nFOG, and HCs. Then, in the second stage, differences in ROI-based FC in patients with MSA with FOG were used to detect DC alterations. DC values were found to vary more widely in patients with MSA-FOG than patients with MSA-nFOG; significantly reduced DC values were detected in the thalamus of patients with MSA-FOG when compared to the MSA-nFOG and HC groups. Furthermore, the mean zDC values for the thalamus were negatively correlated with FOGQ scores. In addition, thalamus-dominated FC analyses indicated that increased thalamus-based FC, including the bilateral cerebellum_8, the right IPL, and the left MTG could provide new insight into the thalamus-dominated pathophysiological mechanism underlying FOG in MSA.

Of the areas showing alterations in DC, only the left thalamus was identified to be positively correlated with the severity of FOG; other DCs showing intergroup differences between the MSA-FOG and MSA-nFOG groups were not correlated with FOGQ scores. These results suggested that the disruption of DC in the thalamus was involved in the pathophysiological mechanism underlying FOG and may serve as a potential neuroimaging marker. In fact, by connecting with the basal ganglia, the cerebellum, and the cortex, the thalamus participates in feedback and feed-forward mechanisms and plays a modulatory role in the integration of information across the parallel motor, cognitive and limbic circuits (Alexander et al., 1986; Haber and McFarland, 2001; Borra et al., 2015; Quartarone et al., 2020). Our results further highlight the importance of the thalamus in MSA patients with FOG symptoms. From the perspective of transmitter disorder, this is easy to explain; on the one hand, normal postural function depends in part on the ability of the postural control system to integrate visual, proprioceptive, and vestibular sensory information. The degeneration of cholinergic neurons in the brainstem pedunculopontine nucleus complex and their thalamic efferent terminals can directly cause postural control deficits (which will induce FOG or fall symptoms) in both PD and MSA diseases (Gilman et al., 2010; Bohnen et al., 2019; Wilson et al., 2021). On the other hand, supported by findings that dopamine therapy is a protective factor in patients with MSA (Yang et al., 2022), we hypothesize that a reduction in dopamine availability or effectiveness in patients with both PD and MSA results in reduced inhibitory action in the thalamic nuclei that trigger thalamo-striatal feedback or thalamo-cortical feed-forward mechanisms during the augmentation of motor output and the indirect induction of FOG symptoms (Nanda et al., 2009), which can be proved by the fact that striatal dopaminergic denervation is critical in the pathophysiology of FOG in MSA, given that it occurs most frequently when patients are in the OFF state and all patients recruited in this study belong to dopamine-responsive FOG variations. Of course, the present fMRI study only indicated that thalamic DC alterations were involved in the regulation of FOG symptoms in patients with MSA. Whether this process involves a disorder in the cholinergic neurons or dopamine transmitter function needs further PET-specific transport receptor research.

It is worth noting that patients with MSA-P and MSA-C subtypes (MSA Parkinsonian and the cerebellar variant) were included in this study. The incidence of FOG was almost equal in the MSA-P and MSA-C subtypes in our study; this finding is inconsistent with previous studies, which found that thalamic injury was more significant in patients with MSA-P (Minnerop et al., 2007; Campabadal et al., 2022; Yang et al., 2022). Furthermore, we found no significant difference between the two subtypes in terms of non-motor symptoms and autonomic nervous function symptoms or when compared between the presence and absence of FOG symptoms. Although MSA-P and MSA-C subtypes were characterized by impairments in the supratentorial basal ganglia and infratentorial cerebellum, respectively, both MSA variants showed dysfunction in the thalamus and cerebellum (Minnerop et al., 2007; Dash et al., 2019); therefore, any dysfunction in the cerebellar-thalamo-cortical (CTC) or striatal-thalamo-cortical (STC) circuits in patients with MSA would be expected to lead to the onset of FOG; this possibility requires further investigation.

Compared to the patients with MSA-nFOG, the patients with MSA-FOG showed a decreased FC between the thalamus and the bilateral cerebellum. Abnormal cerebellar processing is expected to reflect FOG symptoms in patients with MSA. The cerebellar processing of proprioceptive information is important in the regulation of ongoing movements and the maintenance of a stable gait (Takakusaki, 2008). The most consistent finding arising from studies of MSA is damage to the cerebellar structure. Previous studies found that cerebellar injury is a clear risk factor for FOG symptoms in patients with MSA (Gurevich and Giladi, 2003) and that the cerebellar locomotor region is responsible for FOG-like symptoms (Fasano et al., 2017). In this study, we first revealed FOG symptoms in patients with MSA by the application of neuroimaging techniques despite indirect focus on the cerebellum; our findings were consistent with previous studies, thus suggesting that the cerebellum could play an important role in the neural mechanisms underlying FOG symptoms in patients with MSA. In addition, increased FC between the thalamus and cerebellum may imply a compensatory role through the CTC circuits in patients with MSA-FOG. Unfortunately, our correlation analysis did not detect a reduction in the cerebellar ZFC value in relation to FOG score. Future studies should involve a larger number of patients and directly compare the changes in cerebellar structure and function between FOG and patients with nFOG; such analysis should identify specific neuroimaging mechanisms in the cerebellum of patients with MSA.

In addition to the thalamus and cerebellum, when compared to patients with MSA-nFOG, the patients with MSA-FOG also showed DC abnormalities in the right superior pole temporal gyrus, left middle temporal gyrus, left anterior cingulum cortex right, inferior orbit frontal gyrus, and the right middle frontal gyrus, as well as thalamus-based FC dysfunction in the left middle temporal gyrus, right inferior parietal lobule, and left precuneus. Both the inferior orbit frontal gyrus and the superior/middle temporal gyrus are known to be more engaged in non-motor (memory and emotional) processing, whereas the middle frontal gyrus, the cingulum cortex, and the inferior parietal lobule belong to the node of default-mode network (DMN). Our current findings add to the body of the literature that supports the fact that the regulation of FOG in patients with MSA depends on non-motor circuits. It is worth noting that patients with MSA-FOG showed reduced DC in the thalamus but an increase in the FC between the thalamus and non-motor cortex, although no specific correlation was detected between the non-motor cortex and clinical FOGQ scores. We hypothesize that the purpose of the increased thalamus-nonmotor circuit FC was to overcome the reduced level of processing in the depleted sensorimotor circuits. Over time, it is likely that altered processing in these compensatory circuits predisposes them for gait breakdown and the onset of FOG.

There were limitations in our study that need to be considered. First, despite a 12 h washout before the scan, the influence of the medication used cannot be fully excluded; the specific effect of levodopa on MSA and the effects of residual dopamine on FOG remain unknown (Nonnekes et al., 2020). Second, as described in the previous studies (Wang et al., 2018; Yang et al., 2020), we only used *r* > 0.25 as a threshold in our study when calculating DC. In the future studies, we aim to include other thresholds for comparison; this may help us to eliminate the influence of methodological choice on experimental results. Third, the clinical symptoms of patients with MSA and patients with spinocerebellar ataxia partially overlap; therefore, to avoid the impact of misdiagnosis on our experimental results, we conducted a series of spinocerebellar ataxia 1, 2, 3, 6, and 7 gene tests (the most common subtypes in the Chinese population) (Wang et al., 2010), and the lack of detection for other genetic subtypes has had an inevitable effect on FOG research in patients with MSA. Comprehensive genetic testing is recommended in the future studies to rule out confounding diseases, such as hereditary and subacute diseases combined with degeneration of the spinal cord. Fourth, the thalamus of each hemisphere can be subdivided into 15 subregions. Each thalamic subregion participates in different functional processes, either individually or collaboratively. Thus, considering the overall functional changes may not reveal panoramic information relating to specific thalamic nuclei; this is a notable limitation of this study. Multicenter prospective cohort studies and longer follow-ups of FOG symptoms might overcome these limitations.

## Conclusion

In this RS-fMRI study, we showed that the thalamus is the hub region for DC alterations in FOG-associated MSA. Behavioral associations and thalamocortical connectivity suggested that the non-motor circuit played a compensatory role in MSA. These findings provide neuroimaging evidence for a better clinical understanding of non-pure depression and may help us to develop new therapeutic strategies.

## Data availability statement

The raw dataset is not publicly available due to the inherently identifiable nature of the data. Requests to access the datasets should be directed to YC, Elim2501436@163.com.

## Ethics statement

The studies involving human participants were reviewed and approved by the Renmin Hospital of Wuhan University Ethics Committee. The patients/participants provided their written informed consent to participate in this study.

## Author contributions

YC, HY, and JC conceived the study. YC, HY, and ZW collected and analyzed the data. WVL contributed to the writing – review and language polishment. JC revised the article and finally approved the version to be submitted. All authors read and approved the final manuscript.

## Footnotes

1
www.restfmri.net
